# Lower limb amputees undergo long-distance plasticity in sensorimotor functional connectivity

**DOI:** 10.1038/s41598-019-39696-z

**Published:** 2019-02-21

**Authors:** Ivanei E. Bramati, Erika C. Rodrigues, Elington L. Simões, Bruno Melo, Sebastian Höfle, Jorge Moll, Roberto Lent, Fernanda Tovar-Moll

**Affiliations:** 1 D’Or Institute for Research and Education (IDOR), Rio de Janeiro, 22281-100 Brazil; 2 Institute of Biomedical Sciences, Federal University of Rio de Janeiro (UFRJ), Rio de Janeiro, 21941-902 Brazil; 3 National Centre for Structural Biology and Bioimaging, Federal University of Rio de Janeiro (UFRJ), Rio de Janeiro, 21941-902 Brazil; 4 Augusto Motta University (Unisuam), Rio de Janeiro, 21041-020 Brazil; 5 Rio de Janeiro State University (UERJ), Rio de Janeiro, 20550-900 Brazil

## Abstract

Amputation in adults is associated with an extensive remapping of cortical topography in primary and secondary sensorimotor areas. Here, we used tactile residual limb stimulation and 3T functional magnetic resonance imaging in humans to investigate functional connectivity changes in the sensorimotor network of patients with long-term lower limb traumatic amputations with phantom sensation, but without pain. We found a pronounced reduction of inter-hemispheric functional connectivity between homologous sensorimotor cortical regions in amputees, including the primary (S1) and secondary (S2) somatosensory areas, and primary (M1) and secondary (M2) motor areas. We additionally observed an intra-hemispheric increased functional connectivity between primary and secondary somatosensory regions, and between the primary and premotor areas, contralateral to amputation. These functional connectivity changes in specialized small-scale sensory-motor networks improve our understanding of the functional impact of lower limb amputation in the brain. Our findings in a selective group of patients with phantom limb sensations, but without pain suggest that disinhibition of neural inputs following traumatic limb amputation disrupts sensorimotor topology, unbalancing functional brain network organization. These findings step up the description of brain plasticity related with phantom sensations by showing that pain is not critical for sensorimotor network changes after peripheral injury.

## Introduction

Amputation in adults is associated with the remapping of sensorimotor cortical representations. Changes in the primary sensorimotor cortical areas in amputees have been described employing different electrophysiological and imaging techniques^1–5^. About 90% of individuals suffering amputation report “phantom sensations”, the term used to define the perception that the missing part is still present^6–9^. Often these phantom sensations include pain. Studies have correlated phantom limb pain to a primary sensorimotor functional remapping after amputation, suggesting a maladaptive plasticity^10,11^. More recently, the widely-accepted hypothesis that maladaptive plasticity is related to phantom pain in amputees has been challenged. An increased activity in the deprived sensorimotor cortical region corresponding to the amputated hand was reported^12,13^. These apparent conflicting results suggest that a more complex and perhaps multifactorial explanation to the phantom phenomena may be at play. Accordingly, cortical plasticity has also been identified in amputees without phantom limb pain^5,14,15^, but non-painful phantom sensations were not consistently related to primary somatosensory cortex changes after amputation^1^.

Structural changes were also demonstrated in amputees. Gray matter reduction within the hand representation in upper limb amputees as well as white matter changes in the corpus callosum in lower limb amputees were identified^12,15^. Taken together, these functional and structural findings suggest that limb amputation lead to marked changes of sensorimotor network as a whole, potentially unbalancing its functional organization^16^.

Brain networks currently can be investigated in greater depth by functional magnetic resonance imaging functional (fMRI) connectivity methods during the performance of specific tasks. These methods can be used to analyse the functional relationships among different brain areas in detail^17,18^ and to identify the contribution or influence of different brain regions to accomplish a specific task or behaviour, and the degree of coupling between components of distributed neural systems^19^. A resting-state approach – during which participants are not engaged with any external stimulus or explicit tasks – was recently employed to show a reduced interhemispheric functional connectivity between the two hand areas in hand amputees compared to controls^12^. Additionally, a reduction in functional connectivity between the missing hand motor representation and the sensorimotor network was observed^11^. However, because these studies investigated resting-state connectivity, it is unknown whether such changes would be present while participants are engaged in sensorimotor tasks. Additionally, these studies focused on the hand representation and its relations in particular and include participants with different pain ratings.

In the current study, we aimed to investigate the connectivity of a specialized network related to somatosensory stimulation in amputees without pain. To achieve this goal, we employed functional connectivity methods to analyse task-based fMRI time series data of lower limb amputees and controls.

## Results

We report extensive functional connectivity changes in lower limb amputees without pain during two tactile stimulations: residual limb stimulation and remaining foot stimulation, as compared to controls. Sensorimotor brain connectivity was investigated using the region of interest (ROI-to-ROI) connectivity method^20,21^. A priori ROIs were: primary motor cortex (M1); premotor cortex/supplementary motor area (M2); primary somatosensory cortex (S1); and secondary somatosensory cortex (S2).

### Functional connectivity changes during residual limb stimulation

**Figure 1:**
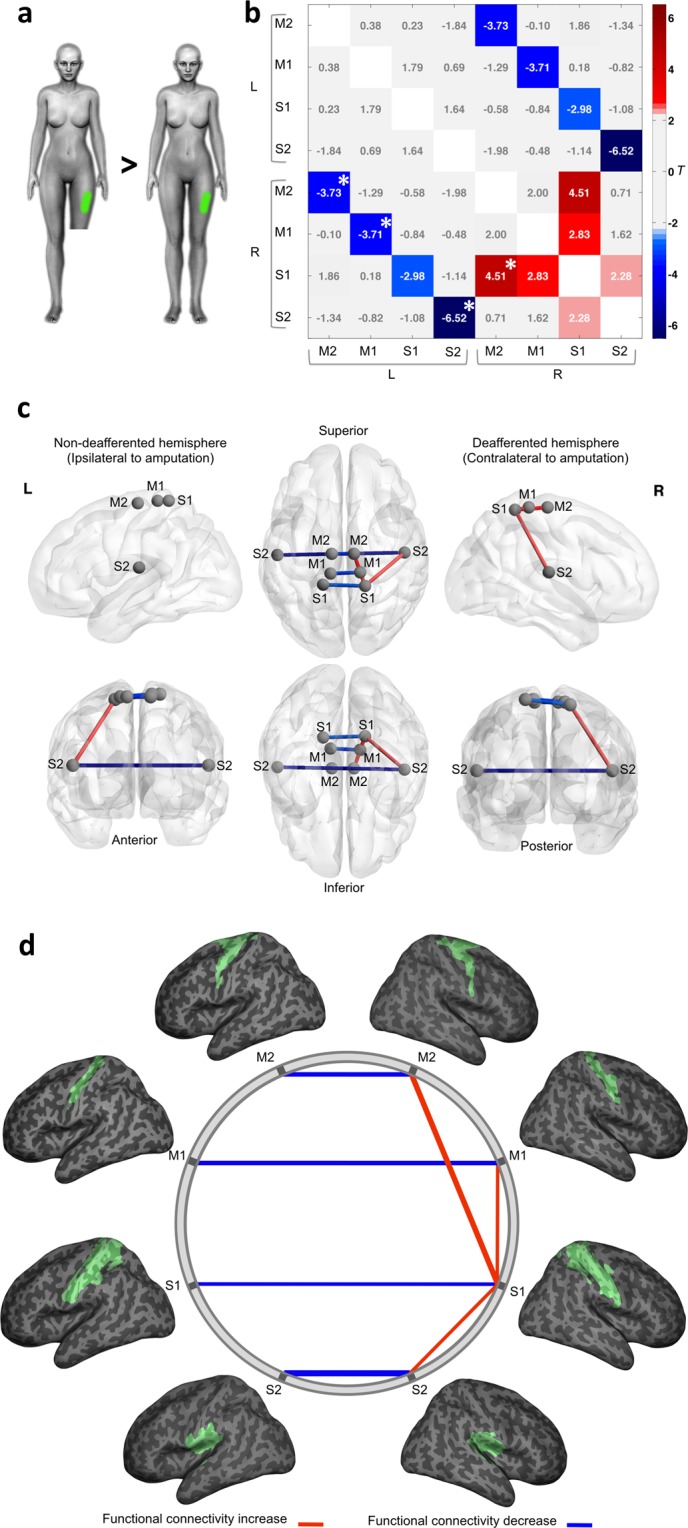
Functional connectivity analysis in the “residual limb” model. (**a**) Schematic representation of the proposed contrasts. In green is an example of the stimulated area in an amputee (left) and his matched control (right). (**b**) The connectivity matrix displays *T* values for the group comparisons in both hemispheres. Significant connections (edges) were thresholded at *P* < 0.05, FDR corrected. Significant FWE corrected values, thresholded at *P* < 0.05, are marked with an asterisk (*). The statistically significant connectivity differences between amputees and controls are highlighted. Positive *T* values are related to significant increased connectivity (red) and negative *T* values are related to significant decreased connectivity (blue) in amputees compared to the control group. (**c**) Graph representation of nodes and significant edges displayed over a MNI stereotactic glass brain 3D-reconstruction, visualized with BrainNet Viewer^73^ (RRID:SCR_009446; http://www.nitrc.org/projects/bnv/). The hemisphere contralateral to amputation (deafferented) is displayed as the right hemisphere, while the hemisphere ipsilateral to amputation (non-deafferented) is displayed at left. (**d**) Schematic representation of the same functional connectivity alterations (network edges of connections). ROIs are shown overlayed onto a 3D inflated brain surface, positioned in stereotaxic coordinates. Red lines represent significantly increased connectivity and blue lines represent significantly decreased connectivity in amputees compared to the control group. The line width is proportional to the *T* values for each statistical contrast.

General Linear Model (GLM) statistical comparisons of ROI-to-ROI functional connectivity during tactile stimulation of the residual limb from the amputees’ group (see Table 1 for detailed clinical characteristics) compared with its homologous region on the control group revealed significant differences, thresholded at *P* < 0.05 for false discovery rate (FDR-corrected) (Fig. 1). Family-wise error values, thresholded at *P* < 0.05 (FWE-corrected), were also calculated. There was an increase in intra-hemispheric functional connectivity contralateral to amputation in the amputees. Ipsilateral connectivity from S1 to M1 (*T* = 2.83, *P*^*FDR*^ = 0.009), S1 to S2 (*T* = 2.28, *P*^*FDR*^ = 0.036), and S1 to M2 (*T* = 4.51, *P*^*FDR*^ = 0.001) were increased in the “deafferented” hemisphere (i.e., contralateral to the amputation) (Table 2). Inversely, a lower inter-hemispheric functional connectivity was found in amputees compared to controls. This decreased functional connectivity was found for all the inter-hemispheric connections between the homotopic regions: S1 (*T* = −2.98, *P*^*FDR*^ = 0.008), M1 (*T* = −3.71, *P*^*FDR*^ = 0.001), M2 (*T* = −3.73, *P*^*FDR*^ = 0.001), and S2 (*T* = −6.52, *P*^*FDR*^ = 0.000) (Table 2).

**Table 1:** Detailed clinical characteristics of amputees.

Subjects	Gender	Age at scan	Age at amputation	Time since amputation	Cause of amputation	Level of amputation	Side	Phantom sensations	Phantom limb
PAC01	F	39	8	31	Traumatic	Transtibial	Right	Foot far from the residual limb; normal size	Permanent
PAC02	F	18	8	10	Traumatic	Transfemoral	Right	Variable; shortened foot and leg	Permanent
PAC03	F	23	13	10	Traumatic	Transtibial	Left	Leg with normal length	Intermittent
PAC04	F	41	20	20	Traumatic	Transfemoral	Left	Thigh and leg in forced posterior flexion and fixed	Permanent
PAC05	M	24	15	8	Traumatic	Transfemoral	Left	Distal foot in plantar flexion	Intermittent
PAC06	M	39	12	27	Traumatic	Transfemoral	Right	Foot far from the residual limb; normal size	Intermittent
PAC07	M	41	17	24	Oncological procedure	Transfemoral	Left	Fully extended leg	Permanent
PAC08	M	33	25	7	Traumatic	Transtibial	Left	Phantom foot and leg	Permanent
PAC09	M	38	19	19	Traumatic	Transfemoral	Left	Phantom ankle and leg	Intermittent

**Table 2:** Summary of differences in functional connectivity between amputees and controls in the **“**residual limb**”** model.

ROI A	ROI B	*T*	*P* ^*FDR*^	*P* ^*FWE*^
S2_L	S2_R	−6.52	0.000	0.000
S1_R	M2_R	4.51	0.001	0.001
M2_L	M2_R	−3.73	0.001	0.044
M1_L	M1_R	−3.71	0.001	0.050
S1_L	S1_R	−2.98	0.008	0.963
S1_R	M1_R	2.83	0.009	0.995
S1_R	S2_R	2.28	0.036	1.000

Differences in functional connectivity between amputees and controls in the “residual limb” model, listed in order of FDR-corrected statistical significance (*P* < 0.05). FWE-corrected values are also shown. Positive *T* values indicate increased connectivity in amputees compared to controls between each pair of ROIs. Negative *T* values indicate decreased connectivity in amputees compared to controls.

### Functional connectivity changes during stimulation of the intact foot

**Figure 2:**
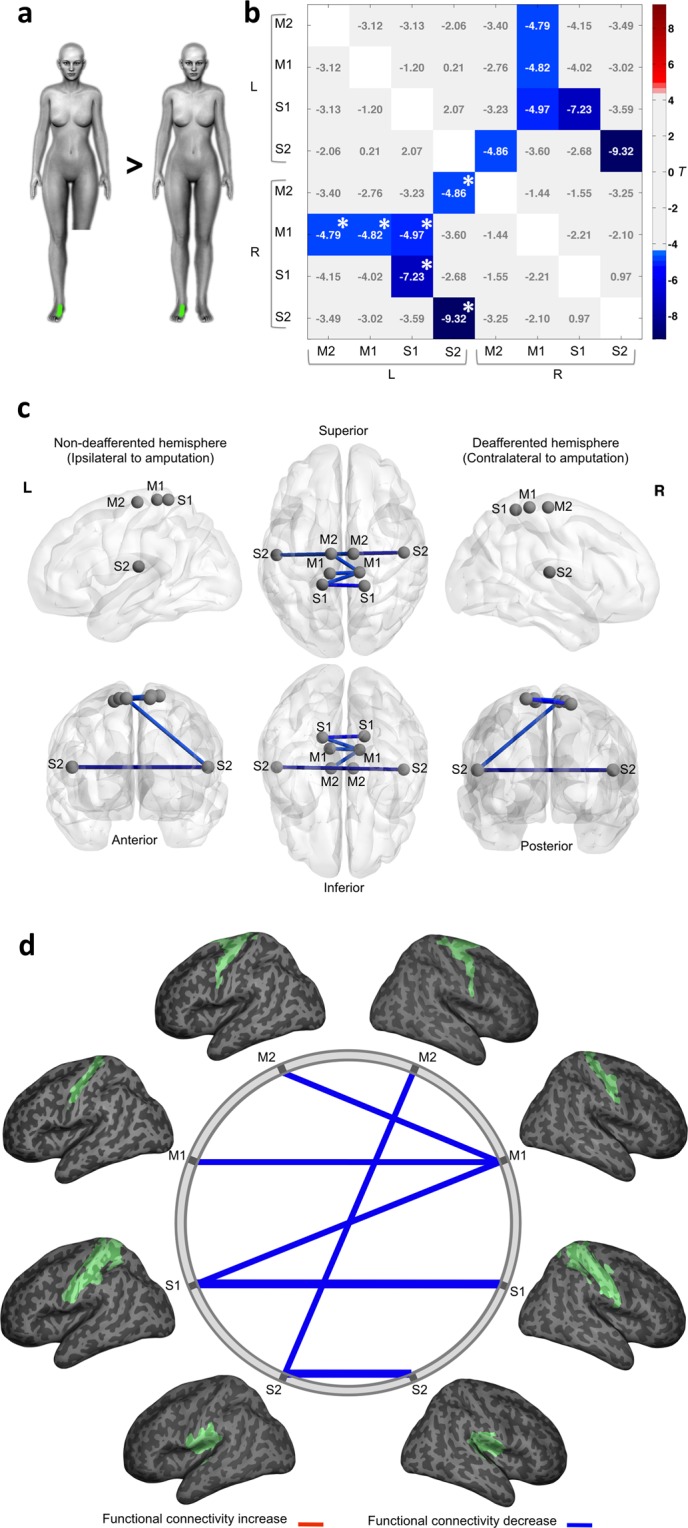
Functional connectivity analysis in the “foot” model. (**a**) Schematic representation of the proposed contrasts. In green is an example of the stimulated area in an amputee (left) and his matched control (right). (**b**) The connectivity matrix displays T values for the group comparisons in both hemispheres. Significant connections (edges) were thresholded at P < 0.05, FDR corrected. Significant FWE corrected values, thresholded at P < 0.05, are marked with an asterisk (*). The statistically significant connectivity differences between amputees and controls are highlighted. Positive T values are related to significant increased connectivity (red) and negative T values are related to significant decreased connectivity (blue) in amputees compared to the control group. (**c**) Graph representation of nodes and significant edges displayed over a MNI stereotactic glass brain 3D-reconstruction, visualized with BrainNet Viewer^73^ (Xia *et al*., 2013; RRID:SCR_009446; http://www.nitrc.org/projects/bnv/). The hemisphere contralateral to amputation (deafferented) is displayed as the right hemisphere, while the hemisphere ipsilateral to amputation (non-deafferented) is displayed at left. (**d**) Schematic representation of the same functional connectivity alterations (network edges of connections). ROIs are shown overlayed onto a 3D inflated brain surface, positioned in stereotaxic coordinates. Blue lines represent significantly decreased connectivity in amputees compared to the control group. The line width is proportional to the T values for each statistical contrast.

Differences in brain connectivity after amputation were not restricted to the affected limb (residual limb stimulation). Functional connectivity during stimulation of the spared foot in amputees was also significantly different from foot stimulation in the control group (Fig. 2). Specifically, statistically significant inter-hemispheric decrease of functional connectivity in homotopic regions was found in amputees: M1 (*T* = −4.82, *P*^*FDR*^ = 0.008), S1 (*T* = −7.23, *P*^*FDR*^ = 0.000), and S2 (*T* = −9.32, *P*^*FDR*^ = 0.000) (Table 3). Additionally, functional connectivity decrease was found between three other inter-hemispheric connections: deafferented M2 with the contralateral S2 (*T* = −4.86, *P*^*FDR*^ = 0.008), and deafferented M1 with the contralateral M2 and S1 (*T* = −4.79, *P*^*FDR*^ = 0.008; *T* = −4.97, *P*^*FDR*^ = 0.006) (Table 3).

**Table 3:** Summary of differences in functional connectivity between amputees and controls in the **“**foot**”** model.

ROI A	ROI B	*T*	*P* ^*FDR*^	*P* ^*FWE*^
S2_L	S2_R	−9.32	0.000	0.001
S1_L	S1_R	−7.23	0.000	0.001
S1_L	M1_R	−4.97	0.006	0.008
S2_L	M2_R	−4.86	0.008	0.010
M1_L	M1_R	−4.82	0.008	0.012
M2_L	M1_R	−4.79	0.008	0.014

Differences in functional connectivity between amputees and controls in the “foot” model, listed in order of FDR-corrected statistical significance (*P* < 0.05). FWE-corrected values are also shown. Negative *T* values denote decreased connectivity in amputees compared to controls between each pair of ROIs.

## Discussion

In the present study, we described functional connectivity changes in the sensorimotor network in lower limb amputees with phantom sensation, but without pain. Remarkably, during somatosensory stimulation, extensive sensorimotor functional connectivity changes were found. A marked reduction of inter-hemispheric functional connectivity between homologous sensorimotor areas was found along with an increased intra-hemispheric connectivity contralateral to the amputated limb.

Typically, normal inter-hemispheric functional connectivity of the brain is characterized by higher symmetrical brain network patterns between homologous areas, as compared to asymmetrical connectivity between heterotopic regions^16^. In accordance the sensorimotor system usually exhibits high levels of inter-hemispheric functional connectivity between homologous brain areas (for a review, see Fabri *et al*., 2014)^22^.

During both residual limb and foot stimulation in the amputees (Figs 1 and 2), however, we found a pronounced reduction of inter-hemispheric functional connectivity between homologous regions along the sensorimotor cortical network in comparison to controls. Connectivity changes had also been observed after peripheral nerve injury. For instance, the inter-hemispheric functional connectivity of the sensorimotor network and between primary motor areas during rest was found to be reduced following brachial plexus avulsion injury^23,24^. Forepaw denervation in animal models also seems to produce connectivity reduction^25^. Furthermore, a study has shown inter-regional connections asymmetry between sensorimotor regions in individuals with congenital upper limb absence^26^.

Weakened inter-hemispheric functional connectivity between hand representations during rest has already been described in upper limb amputees^11^. Nevertheless, the statistical effect observed by Makin and co-workers^11^ was lost when the phantom limb pain magnitude was accounted in the model. Although this result reinforces the idea that phantom limb pain can influence sensorimotor system organization, it also suggests a potential additive effect of brain changes related to limb amputation - as observed in the present investigation - and the ones related to the presence of pain per se.

In non-human primates, electrophysiological studies associated with anatomical tracer injections have demonstrated a predominantly homotopic connection between S1, S2 and M1 of both hemispheres via the corpus callosum (CC)^27–29^. Additionally, this same circuitry has been recently described in humans through magnetic resonance imaging (MRI) and electroencephalography (EEG) approaches^22,30,31^. Considering the importance of transcallosal connection between sensorimotor areas of both hemispheres^28,32,33^, the functional connectivity decoupling found here could be related to fractional anisotropy reduction in the CC previously reported in the same group of patients^15^. Despite containing mostly excitatory fibres that contact the opposite hemisphere^34–36^, the net result of CC activity is importantly inhibitory, mainly intermediated by GABAergic interneurons^37–40^. Considering this inhibitory role, a reduction in the integrity of the corpus callosum in amputees should probably lead to a decrease of functional connectivity between the two hemispheres, as indeed was found in the present work. Other structural findings also suggest that inter-hemispheric relationships can be changed after amputation. For instance, S1 cortex thickness between the two hemispheres did not correlate in lower limb amputees as it does in controls^41^.

Functionally, a reduction in inter-hemispheric inhibition may also help explain the bilateral somatosensory cortex activation found during somatosensory stimulation of the residual limb and the contralateral foot previously reported in the same group of amputees^15^. Animal models support the existence of long-range inter-hemispheric cortical reorganization, probably mediated by the CC. In rodents, unilateral hind limb denervation leads to bilateral functional activation of the primary somatosensory cortex during sensory stimulation of the intact hind limb. This ipsilateral activation, however, no longer exists after ablation of the cortex contralateral to the healthy paw, highlighting the role of CC projections to this phenomenon^42^. In addition, microstructural cortical changes in callosal axons were also recently reported in a neonate rat model of amputation^43^. Is this study, the callosal axons originated from the deafferented S1 area expressed an expansion of their terminal arbors and an increase of the number of terminal boutons within the homotopic representation (S1) at the contralateral cerebral hemisphere.

During tactile residual limb stimulation, along with interhemispheric connectivity reduction, we have found an increase of functional intra-hemispheric connectivity between primary and secondary somatosensory regions, and also between primary and premotor/supplementary motor areas of the hemisphere contralateral to amputation. Conceivably, this increase in functional connectivity between areas within the same hemisphere may be related to the augmented cortical activation during stimulation of the residual limb and in the intact foot previously reported in amputees^15^.

Functional expansion of the cortical representation of remaining body parts into those of deprived sensory input has already been demonstrated in different animal models^43–45^ as well as in humans^46^. This expansion can parallel an increase of the intra-hemispheric functional connectivity and might be related to the unmasking of cortical representations, inhibited under normal conditions^47^.

In fact, the unmasking of pre-existent projections is a potential mechanism for the increases observed in intra-hemispheric connectivity. Previous work with animal models has shown functional connectivity between primary somatosensory barrel cortex and other cortical areas in normal mice^48–50^. By using voltage-sensitive dye imaging, Ferezou and his colleagues^49^, have shown that after a stimulus delivered onto a single mouse whisker, a response could be seen primarily at the corresponding S1 barrel cortex and then spread across S2 and M1 with small latency. In addition, if the S1 barrel cortex was inactivated by an ionotropic glutamate receptor antagonist, no response could be recorded in neither S1 nor any other cortical area^49^. These findings indicate a direct connection between S1 and different motor areas of the cortex.

In animal models, anatomical connectivity can be studied by directly injecting tracers into specific brain regions^43,45,51^. Indeed, experiments with anatomical tracers have shown a direct anatomical and reciprocal connection between S1 and M1, and between S1 and perirhinal cortex of the same hemisphere in normal mice^33^. The modulation of these connections may correspond, at least in part, to the underpinning mechanism of the increased intra-hemispheric sensorimotor functional connectivity seem in amputees in the present study.

As an alternative explanation of our results, we could speculate about subcortical mechanisms that could account for it. Somatosensory cortical areas have extensive long-range connectivity with subcortical nuclei along the somatic pathway^33,51^. Using tracers and also S1 and S2 focal cortical lesions in rats with forelimb amputation, Pearson and colleagues^51^ suggest a major role of subcortical sites in cortical reorganization. Accordingly, one could hypothesize a disinhibition of the anatomical connections between the residual limb area in somatosensory brainstem nuclei and the leg and foot areas in the thalamus, produced by amputation. This disinhibition could make the leg and foot representation areas in the thalamus become responsive to an input from the residual limb area, and then relay this input to the leg and foot areas in S1. Thus, when the residual limb is stimulated, this input would be delivered to the leg and foot representation areas in addition to the corresponding S1 area. Therefore, residual limb stimulation would activate more cortical areas than a stimulus made in an equivalent area in control subjects. In accordance to this proposal, thalamic mapping with microelectrodes in humans revealed an enlargement of the residual limb representation in amputees^52^. Aronoff and colleagues^33^ suggested that S1 has a functional and anatomical connection with motor areas; consequently, an increase of the number of cortical areas activated by a stimulus could lead to an increase of functional connectivity between S1 and S2, S1 and M1, and S1 and M2. All those findings are in agreement with our intra-hemispheric connectivity results.

In this study, we selected patients with phantom limb sensation but who did not report phantom pain (five with permanent phantom sensations and four with intermittent phantom sensations). Whether there are functional connectivity differences between amputees without phantom limb pain who have permanent vs. intermittent phantom sensation is an important question that was beyond the scope of our study and remains unanswered. We believe that the perception of phantom limb sensation, whether intermittent or permanent, should play an important role in altering the balance of the sensorimotor cortical networks. The investigation of these possible differences can be the object of future studies. Furthermore, one of the main limitations of this study was the lack of a specific validated scale to quantify the phantom sensation reported by each of the amputees. Therefore, it was not possible to make any assumption about the relationship between the degree of phantom sensation and connectivity changes.

To conclude, our results of functional connectivity in long term lower limb amputees with phantom sensations, but without pain, advance the current knowledge about brain correlates of phantom sensations by showing sensorimotor network changes pointing to interhemispheric decrease and intrahemispheric increase between the connected cortical areas.

## Materials and Methods

### Participants

This study investigated functional connectivity in nine patients (5 men; averaging 32.9 years/old; range 18–41 years) with unilateral lower limb amputation (8 traumatic amputees and one amputated after an oncologic procedure) with phantom limb sensation and without pain, selected from the Amputee Unit Database of the Brazilian Beneficent Rehabilitation Association (ABBR, Rio de Janeiro). Exclusion criteria for the study included: presence of phantom pain and/or amputation residual limb pain; amputation caused by peripheral obstructive vascular disease; diabetes mellitus with associated peripheral neuropathy; arterial hypertension; renal disfunction; history of neurological disease; neurotrauma; neurosurgical intervention or cerebrovascular disease; use of psychotropic, neuroleptic, anxiolytic or anti-depressant medication; and any contraindication to magnetic resonance imaging. A trained neurologist collected the clinical history of all subjects. The anamnesis included questions about the circumstances of amputation and a detailed assessment of phantom sensations, encompassing the time of onset, the pattern (intermittent/permanent), and the presence of pain, among others. The characteristics of the amputees were reported in a previous study^15^ and are summarized here in Table 1. Nine healthy age- and gender-matched volunteers were recruited as a control group (aged 31.6 ± 9.1 years [mean ± SD] ranging from 18 to 41 years). All participants signed an informed consent form before participation. The protocol was approved by the local Ethics Committee (Copa D’Or Hospital/Instituto D’Or de Pesquisa e Ensino, IDOR; no 171/08) and was performed according to Declaration of Helsinki^53^.

### fMRI acquisition

Images were acquired on a 3T magnetic resonance scanner (Achieva, Philips Medical Systems), equipped with a high-performance gradient system (amplitude, 80 mT/m; slew rate, 200 mT/m/ms) and eight-channel sensitivity encoding head coil (parallel acquisition mode). fMRI images based on the blood oxygenation level-dependent (BOLD) signal were acquired with a T2*-weighted, single-shot, fast-field-echo, echo-planar imaging sequence (TR/TE, 2000/35 ms; isotropic voxel size, 3 mm^3^; field-of-view, 230 mm; 24 slices in the axial plane). Foam padding and straps over the forehead and under the chin were used to restrict head motion during acquisition. An fMRI task-based approach was performed using a block design model, with two experimental conditions (rest and somatosensory stimulation): for each condition, 10 dynamic volumes (20 seconds) were acquired 5 times, totalling 100 dynamic volumes per acquisition (3 minutes and 40 seconds of duration). The participants were instructed to stay relaxed and still during the experiment. The amputees received a somatosensory stimulation applied 2 cm above the residual limb end, and over the dorsal aspect of the remnant hallux and foot (“intact foot”). Matched controls received the stimulation on the same region corresponding to the level of amputation (“control residual limb homologue”) and over the dorsal aspect of both hallux and feet (“control foot”). The somatosensory stimulation was a non-painful cutaneous tactile stimulation (5–6 Hz), employing a soft brush over a skin area of 10 × 2 cm applied manually by the experimenter^54^. To allow statistical comparisons, images from three right side amputees and their matched controls were left-right flipped before image processing steps were taken. This procedure has been previously used successfully by other investigators^55^. The hemisphere contralateral to amputation (“deafferented”) is therefore always displayed as the right hemisphere, while the hemisphere ipsilateral to amputation (“non-deafferented”) is displayed as the left hemisphere.

### Image processing

The fMRI data was processed using Statistical Parametric Mapping (SPM8; RRID:SCR_007037; http://www.fil.ion.ucl.ac.uk/spm/). SPM Anatomy toolbox (RRID:SCR_013273; http://www.fz-juelich.de/ime/spm_anatomy_toolbox) was used to extract anatomical regions of interest. Connectivity Toolbox (RRID:SCR_009550; http://www.nitrc.org/projects/conn) was used for temporal processing and functional connectivity analysis. For each subject, the skull and other non-brain tissue were removed from the high-resolution T1 anatomical images using Brain Extraction Tool (BET; RRID:SCR_014586; https://fsl.fmrib.ox.ac.uk/fsl/fslwiki/BET)^56,57^ and the extracted brain was used later on corregistration and normalization steps. The fMRI processing steps includes motion correction (6-parameter rigid body) and spatial smoothing (4 mm Gaussian filter kernel)^58^. For each subject, an averaged functional volume was created to corregister with high-resolution anatomical data. Then, a non-linear normalization between each subjects’ brain and the ICBM152 high resolution (1 mm isotropic voxels) anatomical brain template was performed (MINC/Atlases; RRID:SCR_005281; http://en.wikibooks.org/wiki/MINC/Atlases). The non-linear anatomical normalization parameters were then applied to each acquired functional data. The resulting functional data in Montreal Neurological Institute (MNI) coordinates was utilized to perform functional connectivity group analysis. Segmentation was performed on the ICBM152 template^59^, obtaining probabilistic maps of gray matter (GM), white matter (WM), and cerebrospinal fluid areas (CSF). Next, the maps where eroded using a morphological erosion mathematical operator (2 mm^3^), in order to minimize potential partial volume effects between them, and binarized, resulting in three binary masks (GM, WM and CSF masks).

### Temporal data processing

To avoid or minimize the impact of spurious correlations between voxels induced by different sources of physiological and non-physiological noises, which in turn could potentially increase the chance of false positives on functional connectivity results, several confounding variables were mitigated and extracted *a posteriori* from acquired functional BOLD signals^20,60,61^. From each subject, the estimated six motion parameters (three rotation and three translation parameters), along with their first derivatives, as well the mean BOLD signals from WM and CSF voxels were selected as temporal confounding factors^62^. Also, task paradigm regression (TPR) procedure was used to remove the experimental cyclic task/condition effects from fMRI block-design acquisition. Thus, all the selected temporal confounding factors was then regressed out from the GM BOLD time series at each voxel, using the component-based noise correction method^61^. Finally, the residual BOLD signals were filtered with a 0.01 Hz high-pass filter, in order to remove excessive low-frequency drifts, which could introduce spurious correlations in the functional connectivity analysis.

### Functional connectivity analysis

Two task-based fMRI stimulation data were used: (1) tactile stimulation of the residual limb from the amputees group compared with its homologous region on control group (defined as “residual limb” connectivity model); and (2) tactile stimulation of the remaining foot in the amputees compared to the corresponding foot stimulation in the control group (“foot” connectivity model). To identify possible differences in sensorimotor brain connectivity in lower limb amputees without pain, as compared to controls, we employed the ROI-to-ROI connectivity method.

### ROIs selection

We addressed functional connectivity changes between primary and secondary sensorimotor cortices. Using SPM Anatomy toolbox^63^, *a priori* anatomical ROIs were defined as follows: (1) primary motor cortex (M1), corresponding to the pre-central gyrus, including Brodmann areas BA4a and BA4p^64^; (2) premotor cortex/supplementary motor area (M2), corresponding to Brodmann area BA6^65^; (3) primary somatosensory cortex (S1), including Brodmann areas BA3a, BA3b, BA1 and BA2^66,67^; and (4) secondary somatosensory cortex (S2), corresponding to parietal operculum OP1, OP2, OP3 and OP4, including Brodmann area BA40^68^. Before functional connectivity analysis, all selected ROIs were segmented and masked by GM map.

### Functional connectivity individual analysis

After the average BOLD time series for each subjects’ ROIs were computed, linear measures of functional connectivity analysis were performed at first-level by calculating the bivariate correlation (Pearson correlation) of BOLD signals between each pair of ROIs (representing the effect size or strength of connection between two ROIs), ranging from −1 (maximum negative correlation) to 1 (maximum positive correlation). For each functional connectivity model (“residual limb” and “foot”), Pearson’s correlation coefficients *r* between all ROIs time courses were calculated, creating a correlation matrix for each subject. Afterwards, the correlation coefficients were converted to normally distributed scores using Fisher’s transform to allow for second-level General Linear Model (GLM) group analysis^20,69^. Thus, the resulting Fisher’s z-transformed correlation coefficients (z-scores) were used as a measure of “total” functional connectivity between ROIs at subject-level.

### Functional connectivity group analysis

Following the calculation of ROI-to-ROI connectivity matrices for each subject, these measures were entered into a second-level random-effect GLM analysis to obtain group differences between amputees and controls^20,70^. Between-subjects’ contrasts of interest were inferred by comparing functional connectivity patterns between the two groups of subjects at connection-level. The resulting connectivity matrix displays *T* values for the group comparisons. False positive control in ROI-to-ROI analysis was applied using false discovery rate^71^ (FDR) correction for multiple comparisons and significant connections were thresholded at *P* < 0.05. Furthermore, family-wise error (FWE) values were calculated between-groups and the FWE-corrected values (thresholded at *P* < 0.05) were presented at Tables 1 and 2 for each functional connectivity model respectively^71,72^.

## Data Availability

The datasets generated during the current study are available from the corresponding author on reasonable request.
